# SARS-CoV-2 Spike Protein Stimulates Macropinocytosis in Murine and Human Macrophages via PKC-NADPH Oxidase Signaling

**DOI:** 10.3390/antiox13020175

**Published:** 2024-01-30

**Authors:** WonMo Ahn, Faith N. Burnett, Ajay Pandey, Pushpankur Ghoshal, Bhupesh Singla, Abigayle B. Simon, Cassandra C. Derella, Stephen A. Addo, Ryan A. Harris, Rudolf Lucas, Gábor Csányi

**Affiliations:** 1 Vascular Biology Center, Medical College of Georgia, Augusta University, Augusta, GA 30912, USA; wahn@augusta.edu (W.A.); faburnett@augusta.edu (F.N.B.); apandey@augusta.edu (A.P.); bsingla@uthsc.edu (B.S.); saddo@augusta.edu (S.A.A.); rlucas@augusta.edu (R.L.); 2 Georgia Prevention Institute, Medical College of Georgia, Augusta University, Augusta, GA 30912, USA; absimon@augusta.edu (A.B.S.); cderella@augusta.edu (C.C.D.); ryharris@augusta.edu (R.A.H.); 3 Department of Pharmacology and Toxicology, Medical College of Georgia, Augusta University, Augusta, GA 30912, USA

**Keywords:** SARS-CoV-2, macrophage, epithelial cell, macropinocytosis

## Abstract

Coronavirus disease 2019 (COVID-19) is an infectious disease caused by severe acute respiratory syndrome coronavirus 2 (SARS-CoV-2). While recent studies have demonstrated that SARS-CoV-2 may enter kidney and colon epithelial cells by inducing receptor-independent macropinocytosis, it remains unknown whether this process also occurs in cell types directly relevant to SARS-CoV-2-associated lung pneumonia, such as alveolar epithelial cells and macrophages. The goal of our study was to investigate the ability of SARS-CoV-2 spike protein subunits to stimulate macropinocytosis in human alveolar epithelial cells and primary human and murine macrophages. Flow cytometry analysis of fluid-phase marker internalization demonstrated that SARS-CoV-2 spike protein subunits S1, the receptor-binding domain (RBD) of S1, and S2 stimulate macropinocytosis in both human and murine macrophages in an angiotensin-converting enzyme 2 (ACE2)-independent manner. Pharmacological and genetic inhibition of macropinocytosis substantially decreased spike-protein-induced fluid-phase marker internalization in macrophages both in vitro and in vivo. High-resolution scanning electron microscopy (SEM) imaging confirmed that spike protein subunits promote the formation of membrane ruffles on the dorsal surface of macrophages. Mechanistic studies demonstrated that SARS-CoV-2 spike protein stimulated macropinocytosis via NADPH oxidase 2 (Nox2)-derived reactive oxygen species (ROS) generation. In addition, inhibition of protein kinase C (PKC) and phosphoinositide 3-kinase (PI3K) in macrophages blocked SARS-CoV-2 spike-protein-induced macropinocytosis. To our knowledge, these results demonstrate for the first time that SARS-CoV-2 spike protein subunits stimulate macropinocytosis in macrophages. These results may contribute to a better understanding of SARS-CoV-2 infection and COVID-19 pathogenesis.

## 1. Introduction

Severe acute respiratory syndrome coronavirus 2 (SARS-CoV-2) is a coronavirus that has killed nearly 7 million people worldwide and continues to pose a massive threat to public health [1]. The main identifier of coronaviruses is the presence of the structural spike glycoprotein that protrudes outwards from the surface of the viral envelope [2]. The SARS-CoV-2 spike protein consists of two subunits, S1 and S2, each of which plays a unique role in viral cell infection. The S1 subunit binds with its receptor-binding domain (RBD) to the host entry receptor angiotensin-converting enzyme 2 (ACE2), while the S2 subunit mediates fusion of the virus to the cell through the actions of various proteases [3,4].

The spread of SARS-CoV-2 primarily occurs via respiratory droplets and aerosols. A major target for the virus is the type 1 and 2 alveolar epithelial cells of the lower respiratory tract [5]. Viral infection in the alveolar space can lead to inflammation and barrier dysfunction, both of which can impair gas exchange within the lungs [6]. Prolonged and severe infection can lead to diffuse alveolar damage (DAD), which is the histological manifestation of severe lung disease and inflammation found in patients succumbing to COVID-19 [7]. Within the alveoli, resident alveolar macrophages are the most abundant immune cell type. These cells act as primary defenders against SARS-CoV-2 infections of the respiratory system [8]. Macrophages enter a hyperinflammatory state after infection with SARS-CoV-2 that may result in a detrimental loop of pro-inflammatory cytokine release, reactive oxygen species (ROS) generation and recruitment of cytotoxic immune cells. This sequence of events can further exacerbate inflammation, alveolar damage, and pulmonary edema [9]. Interestingly, studies have shown that SARS-CoV-2 infection in alveolar macrophages may enhance the propagation of the virus across the lung through mechanisms that remain to be identified [10].

Although ACE2 is considered the primary binding site and the receptor responsible for SARS-CoV-2 entry, alternative ACE2-independent internalization pathways, such as macropinocytosis, have also been suggested [11,12]. Macropinocytosis is a receptor-independent mode of endocytosis that leads to the internalization of extracellular fluid and solutes [13]. Macropinocytosis is initiated by the formation of plasma membrane protrusions. These protrusions can develop into plasma membrane ruffles that fold, circularize, and close, forming intracellular vacuoles known as macropinosomes [14]. Previous studies have identified macropinocytosis as an entry mechanism for several viruses including HIV-1 [15], Ebola virus [16], and herpes simplex virus 1 (HSV1) [17]. However, the role of macropinocytosis in SARS-CoV-2 infection and COVID-19 pathogenesis remains poorly understood.

Previous studies demonstrated that macropinocytosis is stimulated via NADPH oxidase 2 (Nox2) in macrophages [18]. NADPH oxidases are a family of membrane-bound enzyme complexes that play a major role in regulating host defense and inflammation. Nox2, the primary isoform found in macrophages, consists of two integral membrane subunits, gp91^phox^ and p22^phox^, and four cytosolic protein subunits: p47^phox^, p67^phox^, p40^phox^, and GTPase Rac [19]. Upon activation, the four cytosolic subunits translocate and bind to the membrane subunits to form the catalytic core of the enzyme. Upstream signal transduction mediators of Nox2, such as PKC and PI3K, have been shown to act as regulators in the formation of membrane ruffles and macropinocytosis [18,20]. While previous studies have identified growth factors, cytokines and phorbol esters that stimulate macropinocytosis via PKC and PI3K signaling, the effect of SARS-CoV-2 spike proteins on the stimulation of this pathway is unknown [21,22,23].

A recent study demonstrated that SARS-CoV-2 utilizes macropinocytosis to enter kidney epithelial and colorectal adenocarcinoma cells [12]. The goal of the present study was to investigate the ability of SARS-CoV-2 spike protein subunits to stimulate macropinocytosis in human alveolar epithelial cells and primary human and murine macrophages.

## 2. Materials and Methods

### 2.1. Reagents

Recombinant SARS-CoV-2 (Wuhan variant) spike protein subunits S1 and S2, as well as the RBD of S1, were purchased from Raybiotech (Peachtree Corners, GA, USA). Human alveolar epithelial cells (HPAEPiC), epithelial cell medium and epithelial cell growth supplement were purchased from ScienCell Research Laboratories (Carlsbad, CA, USA). Mouse and human recombinant macrophage colony-stimulating factor (M-CSF) were obtained from PeproTech (Rocky Hill, NJ, USA). BD Vacutainer CPT tubes were purchased from Fisher Scientific (Waltham, MA, USA). RBC lysis buffer, penicillin–streptomycin, thioglycollate medium FITC-dextran, TRITC-dextran, and H_2_DCFDA were purchased from ThermoFisher (Waltham, MA, USA). RPMI-160 media was purchased from Cytiva (Marlborough, MA, USA). Fetal bovine serum was purchased from GeminiBio (West Sacramento, CA, USA). Calphostin c, LY294002, 5-(*N*-ethyl-*N*-isopropyl) amiloride (EIPA), diphenyleneiodonium chloride (DPI), polymyxin b, and phorbol 12-myristate 13-acetate (PMA) were purchased from Sigma-Aldrich (St. Louis, MO, USA). GSK2795039 was purchased from Tocris Biosceince (Minneapolis, MN, USA).

### 2.2. Cell Culture

The cells were cultured in humidified 5% CO_2_ at 37 °C using the appropriate culture medium. Mouse bone-marrow-derived macrophages were cultured in DMEM medium containing 10% FBS, 100 IU/mL of penicillin G and 100 µg/mL streptomycin. Primary human peripheral blood mononuclear cell (PBMC)-derived macrophages were cultured in RPMI-1640 medium containing 10% FBS, 100 IU/mL of penicillin G and 100 µg/mL streptomycin. HPAEPiC were cultured in alveolar epithelial cell medium containing 2% FBS, 1% epithelial cell growth supplement, 100 IU/mL of penicillin G and 100 µg/mL streptomycin. Bone-marrow-derived monocytes were differentiated into macrophages using murine M-CSF (20 ng/mL, 6 days) [24]. Primary human PBMCs were differentiated into macrophages using human M-CSF (50 ng/mL, 6 days) [25]. Macrophages were incubated in M-CSF free media for at least 1 day prior to experiments.

### 2.3. Primary Human Macrophages

All human study protocols were approved by the Institutional Review Board at Augusta University (#1595933). Human whole blood from four healthy male individuals between the ages 25 and 35 was provided by Dr. Ryan Harris (Georgia Prevention Institute, Augusta University, Augusta, GA, USA). Approximately 16 mL of whole blood from each subject was drawn into 2 BD Vacutainer CPT tubes and centrifuged at 1600× *g* for 20 min at 20 °C. PBMCs were collected and incubated with 1× RBC lysis buffer for 10 min to remove contaminating red blood cells. Cells were then washed with PBS and placed in a −80 °C freezer. Cryopreserved PBMCs were cultured and differentiated to macrophages as described above.

### 2.4. Animals

The mice were housed in accordance with the National Institutes of Health (NIH) guidelines in our AAALAC-accredited experimental animal facility in a controlled environment. All mouse studies were approved by the Institutional Animal Care and Use Committee at Augusta University. For our experiments, we used 12–16-week-old male and female mice weighing between 25 g and 35 g. *C57BL/6J* wild-type (stock #000664) and *LysMCre* (stock #019096) mice were purchased from The Jackson Laboratory (Bar Harbor, ME, USA). *Nhe1^f/f^* mice were kindly provided by Dr. Dandan Sun (University of Pittsburgh, Department of Neurology, Pittsburgh, USA). Male and female *LysMCre^+^ Nhe1^f/f^* (NHE1^∆M^) mice were generated by crossing *Nhe1^f/f^* mice with *LysMCre* mice. Littermate *LysMCre^-^ Nhe1^f/f^* mice were used as controls. All mice were genotyped via PCR amplification of tail DNA. Every effort was made to minimize animal suffering and reduce the number of animals used. The mice were anesthetized (isoflurane inhalation, 3%) prior to bone marrow and peritoneal macrophage isolation.

### 2.5. Isolation of Murine Macrophages

#### 2.5.1. Thioglycollate-Elicited Peritoneal Macrophages

Thioglycollate medium (3%, 1 mL/30 g) was administered via intraperitoneal injection (i.p.) into 12–16-week-old male *LysMCre^+^ Nhe1^f/f^* (NHE1^∆M^) and *LysMCre^−^ Nhe1^f/f^* (NHE1^f/f^) mice. After four days, 1 mg/30 g body weight of FITC-dextran and 20 µg/30 g body weight of spike protein S1 or vehicle (PBS) were injected i.p. into each mouse. After four hours, the mice were anesthetized (isoflurane inhalation, 3%) and euthanized via cervical dislocation. Peritoneal cells were collected using 10 mL of PBS and plated into tissue culture plates. Three hours later, non-adherent cells were removed. Adherent macrophages were washed with PBS and collected using a cell scraper. FITC-dextran internalization was quantified using flow cytometry (excitation 493 nm, emission 518 nm).

#### 2.5.2. Bone-Marrow-Derived Macrophages

We anesthetized and euthanized through cervical dislocation 12–16-week-old female wild-type NHE1^∆M^ and NHE1^f/f^ mice weighing between 25 g and 35 g. The femurs and tibias were cleaned of skeletal muscle tissue, rinsed with 70% ethanol, and placed in sterile PBS. Both ends of each bone were cut and bone marrow cells were flushed out using a 25-gauge needle syringe filled with sterile PBS. Bone-marrow-derived monocytes were differentiated into macrophages using M-CSF as described above.

### 2.6. 2′,7′-Dichlorodihydrofluorescein Diacetate (H_2_DCFDA) Assay

The cell-permeant 2’,7’-dichlorodihydrofluorescein diacetate (H_2_DCFDA) fluorescein (5 µM, ThermoFisher, Waltham, MA, USA) was used to determine intracellular ROS production in macrophages. The cells were treated with the SARS-CoV-2 S1 subunit (1 µg/mL, 1 h) and incubated with H_2_DCFDA (5 µM, 30 min). DCF fluorescence was measured via flow cytometry.

### 2.7. Flow Cytometry

**Figure 1:**
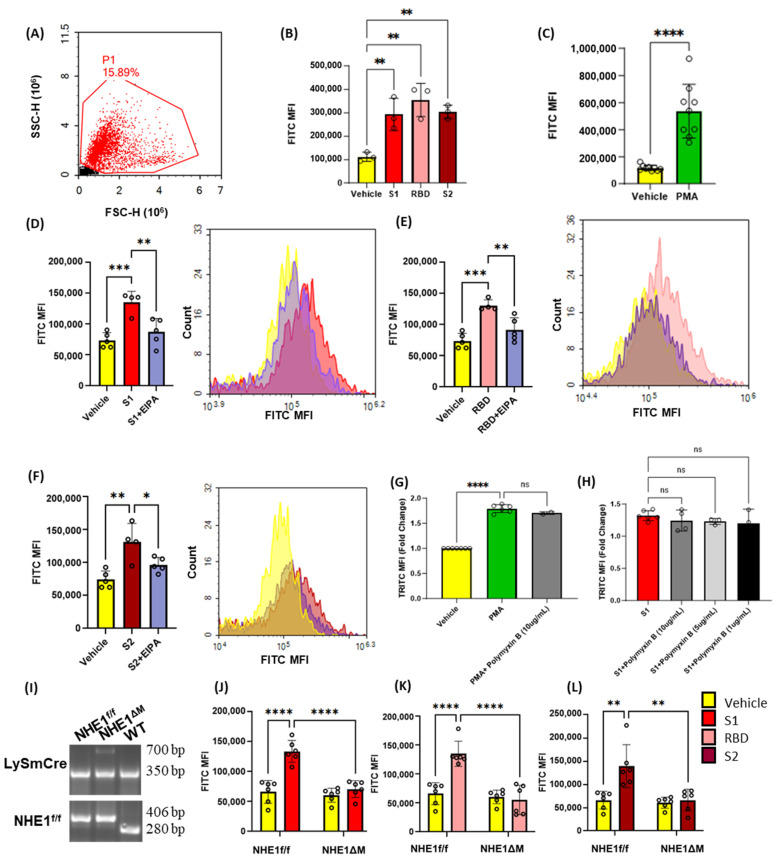
Recombinant SARS-CoV-2 spike proteins stimulate fluid-phase macropinocytosis in murine bone marrow-derived macrophages. (**A**) Flow cytometry gating strategy. (**B**) Murine bone marrow-derived macrophages were incubated with FITC-dextran (100 µg/mL) and treated with vehicle (PBS) or with 1 µg/mL of the spike protein subunits S1, RBD, or S2 for 4 h (n = 3). (**C**) Cells were treated with vehicle or PMA (1 µM, 4 h) in the presence of FITC-dextran (n = 9). Internalization of FITC-dextran was quantified via FACS (ex. 493 nm, em. 518 nm). Cells were pretreated with EIPA (25 µM) prior to incubation with 1 µg/mL of S1 (**D**), RBD (**E**), or S2 (**F**) (n = 4). (**G**) Murine bone marrow-derived macrophages were pretreated with polymyxin b (10 µg/mL, 30 min preincubation) and stimulated with vehicle (PBS) or PMA (1 µM, 4 h) in the presence of TRITC-dextran (100 µg/mL). (**H**) Cells were pretreated with polymyxin b (1–10 µg/mL, 30 min preincubation) and stimulated with S1 subunit (1 µg/mL, 4 h) in the presence of TRITC-dextran (100 µg/mL). Internalization of FITC-dextran was quantified via FACS (ex. 550 nm, em. 577 nm). (**I**) Agarose gel showing representative genotyping experiments. (**J**–**L**) Bone marrow macrophages from NHE1^f/f^ and NHE1^ΔM^ mice were treated with vehicle, S1, RBD, or S2 spike protein subunits (1 µg/mL, 4 h) in the presence of FITC-dextran (100 µg/mL) (n = 6). Data are presented as means ± SD. ns = not significant. * *p* < 0.05; ** *p* < 0.005; *** *p* < 0.001; **** *p* < 0.0001. P values were calculated using *t*-test (**C**), oneway (**B**, **D**–**H**) or two-way (**J**–**L**) ANOVA with Tukey’s test for multiple comparisons.

Flow cytometry experiments were performed using the Acea 4-Laser NovoCyte Quanteon flow cytometer (Agilent, Santa Clara, CA, USA). Each sample was analyzed for 10,000 cells. Macrophages and HPAEPiC were treated with vehicle, spike protein subunits S1, RBD, and S2 (0.001–10 µg/mL), macropinocytosis stimulator PMA (1 µM) and FITC-dextran (70 kDa, fluid-phase marker). Macropinocytosis was inhibited by pre-incubation of cells with EIPA (25 µM, 30 min). In separate experiments, murine bone-marrow-derived macrophages were pretreated with the PKC inhibitor calphostin c (1 µM, 30 min) or PI3K inhibitor LY294002 (10 µM, 30 min), and stimulated with the S1 spike protein subunit (1 µg/mL, 4 h) in the presence of FITC-dextran (100 µg/mL). Additionally, human PBMC-derived macrophages were stimulated with S1 subunit (1 µg/mL, 4 h) ± LL37 (5 µg/mL and 10 µg/mL, 30 min pretreatment). In another experiment, murine bone-marrow-derived macrophages were pretreated with polymyxin b, a lipopolysaccharide inhibitor, at various concentrations (1 µg/mL–10 µg/mL, 30 min) and stimulated with S1 subunit in the presence of TRITC-dextran (100 µg/mL). Finally, HPAEPiC were treated with EGF (0.5 nM, 4 h) ± EIPA (25 µM, 30 min pre-incubation). The cells were washed with PBS, collected and analyzed to determine FITC- or TRITC-dextran internalization using flow cytometry (FITC: 488 nm, band pass filter: 530/30; TRITC: 561 nm, band pass filter: 586/20). The cells were gated to exclude dead cells based on the FSC/SSC plot as shown in Figure 1A. The experimental groups were compared to the vehicle controls (FITC-dextran or TRITC-dextran only). A cell-only group was used as negative controls and cells stimulated with PMA were used as positive controls (Figure 1C). Quantification was determined by assessing the mean fluorescence intensity (MFI) of FITC or TRITC of 10,000 events in each group. T-tests or one-way/two-way ANOVA with Tukey’s test for multiple comparisons were used to determine the significance levels between groups.

### 2.8. Scanning Electron Microscopy

Human THP1 macrophages were treated with vehicle or spike protein subunit S1 (1 µg/mL, 10 min) ± EIPA (25 µM, 30 min pre-incubation). HPAEPiC were treated with 1 µg/mL or 10 µg/mL spike protein subunit concentration. The cells were plated on coverslips at a density of 1 × 10^5^, treated, and fixed (4% PFA, 2% glutaraldehyde in 0.1 M sodium cacodylate solution) overnight at 4 °C. After undergoing a series of dehydration steps using increasing concentrations of ethanol (25–100%), the cells underwent a critical point drying step (Tousimis Samdri-790, Rockville, MD, USA). The cells were sputter coated with 3.5 nm of gold palladium (Anatek USA-Hummer, Sparks, NV, USA) and imaged at 20 KV using a Philips XL30 scanning electron microscope (Hillsboro, OR, USA). The number of membrane ruffles in macrophages and epithelial cells were normalized to the cell number in the microscopic field evaluated.

### 2.9. Statistical Analysis

All data are presented as means ± SD. The data were analyzed using GraphPad Prism 10.1.2 software (GraphPad Software Inc., Boston, MA, USA). Comparisons between groups were analyzed using a t-test or one/two-way analysis of variance (ANOVA) with Tukey’s post hoc test. *p* < 0.05 was defined as statistically significant.

## 3. Results

### 3.1. Recombinant SARS-CoV-2 Spike Proteins Stimulate Macropinocytosis in Murine Macrophages

Although SARS-CoV-2 has been shown to enter kidney epithelial cells via macropinocytosis [12], its ability to stimulate macropinocytosis in cell types directly relevant to COVID-19 pneumonia has not been demonstrated. To investigate the ability of SARS-CoV-2 spike protein subunits to stimulate macropinocytosis in macrophages, we first performed a series of flow cytometry experiments aimed at quantifying spike protein-induced FITC-dextran (70 kDa, fluid-phase marker) internalization in wild-type (*C57Bl/6J*) murine bone-marrow-derived macrophages. Isolated bone-marrow-derived monocytes were differentiated into macrophages using murine M-CSF for 6 days and incubated in M-CSF free media for 1 day prior to experiments. The macrophages were incubated with FITC-dextran (100 µg/mL, 4 h), treated with spike protein subunit S1 (Val16-Gln690), RBD (Arg319-Phe541), or S2 (Met697-Pro1213) (0.001–10 µg/mL, 4 h), and processed for flow cytometry to quantify the degree of FITC-dextran internalization (Supplementary Figure S1). Both spike protein subunits (S1 and S2), as well as the RBD of S1, significantly stimulated FITC-dextran internalization at concentrations of 1 µg/mL (Figure 1A,B). The protein kinase C (PKC) activator phorbol 12-myristate 13-acetate (PMA), which stimulates macropinocytosis in macrophages, was used as a positive control in our experiments (Figure 1C) [26]. Pharmacological inhibition of Na^+^-H^+^ exchanger 1 (NHE1) using amiloride and its more selective analogue 5-(*N*-ethyl-*N*-isopropyl) amiloride (EIPA) is the current gold-standard method to block macropinocytosis in vitro and in vivo [27,28]. As shown in Figure 1D–F, preincubation of macrophages with EIPA (25 µM, 30 min) inhibited FITC-dextran internalization, suggesting that FITC-dextran uptake following spike protein subunit stimulation occurs via macropinocytosis. Because FITC-dextran is pH sensitive, TRITC-dextran (70 kDa) was also used to confirm spike-protein-induced macropinocytosis stimulation (Figure 1G,H). Additionally, because these spike proteins were derived from E. coli, the macropinocytosis-stimulating effect of S1 subunit was tested in the presence and absence of polymyxin b, a potent lipopolysaccharide inhibitor (Figure 1G,H). To confirm the results of this pharmacological approach, we selectively isolated macrophages from mice lacking NHE1 in myeloid cells (*LysmCre^+^ Nhe1^f/f^*, hereafter referred to as NHE1^ΔM^ [28]) and investigated the ability of spike protein subunits to stimulate macropinocytosis. S1, RBD and S2 stimulated macropinocytosis in NHE1^f/f^ controls, but not in NHE1^ΔM^ macrophages (Figure 1I–L). Taken together, these results suggest that SARS-CoV-2 spike protein subunits stimulate macrophage macropinocytosis in vitro.

### 3.2. SARS-CoV2 Spike Protein Subunits Stimulate Macropinocytosis in Primary Human Macrophages

**Figure 2:**
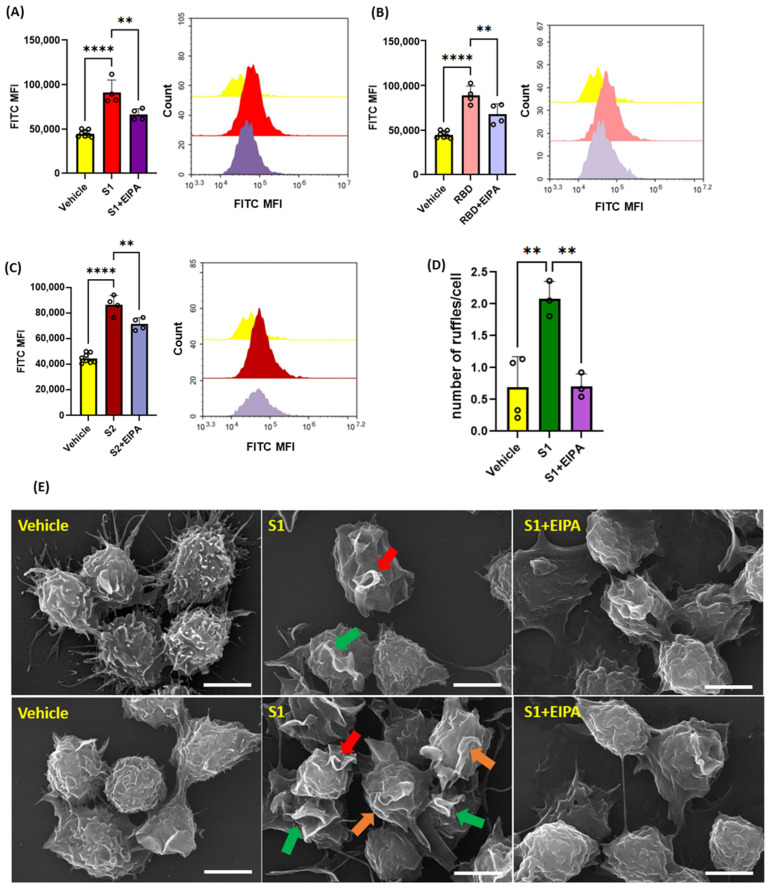
Recombinant SARS-CoV-2 spike proteins stimulate fluid-phase macropinocytosis in human macrophages. Primary human PBMC-derived macrophages were incubated with FITC-dextran (100 µg/mL) and treated with vehicle (PBS) or spike protein subunits S1 (**A**), RBD (**B**), and S2 (**C**) (1 µg/mL, 4 h) ± EIPA (25 µM, 30 min preincubation). FITC-dextran internalization was analyzed via FACS. (**D**) Quantification of the number of ruffles per cell in vehicle-, S1-, and S1+EIPA-treated THP1 macrophages normalized to total cell number. (**E**) THP1 macrophages were treated with spike protein subunits (1 µg/mL, 30 min) ± EIPA (25 µM, 30 min preincubation) and processed for SEM. Analysis of SEM images demonstrated emerging membrane projections (orange arrows), fully formed dorsal ruffles (green arrows), and circularized membrane protrusions forming cups (red arrow). Data are presented as means ± SD. ** *p* < 0.005; **** *p* < 0.0001. P values were calculated using one way ANOVA with Tukey’s test for multiple comparisons. Scale bars are 5 µm.

Next, we investigated the ability of recombinant SARS-CoV2 spike protein subunits to stimulate macropinocytosis in primary human peripheral blood mononuclear cell (PBMC)-derived macrophages. As shown in Figure 2A–C, spike protein subunits S1, S2, as well as RBD of S1 (1 µg/mL, 4 h), stimulated FITC-dextran internalization, while pretreatment with EIPA (25 µM, 30 min) blunted this effect. Macropinocytosis begins with membrane ruffling, followed by macropinocytotic cup formation and internalization of extracellular fluid and associated solutes via the scission of closed macropinosomes from the plasma membrane [14]. We next evaluated whether spike protein subunits stimulate membrane ruffling in human THP1 macrophages using scanning electron microscopy (SEM). Macrophages treated with spike protein S1 (1 µg/mL, 30 min) exhibited marked cell surface ruffling (Figure 2D,E). Emerging membrane projections, fully formed dorsal ruffles, and circularized membrane protrusions forming cups were visualized. Consistent with the flow cytometry experiments, pretreatment with EIPA (25 µM, 30 min) inhibited spike protein stimulation of plasma membrane ruffle formation in human macrophages (Figure 2D,E). These results demonstrate that SARS-CoV-2 spike protein subunits stimulate membrane ruffle formation and macropinocytosis in human macrophages in vitro.

### 3.3. SARS-CoV-2 Spike Proteins Do Not Stimulate Macropinocytosis in Human Alveolar Epithelial Cells

**Figure 3:**
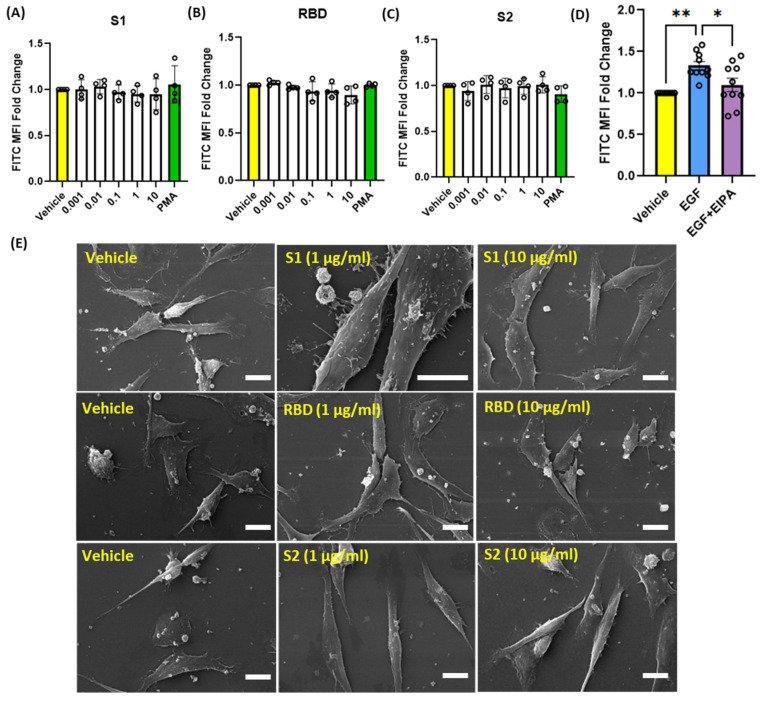
Recombinant SARS-CoV-2 spike proteins do not stimulate fluid-phase macropinocytosis in human alveolar epithelial cells. Cells were incubated with FITC-dextran (100 µg/mL) and treated with vehicle (PBS) or with different concentrations (0.001 µg/mL–10 µg/mL) of the spike protein subunits S1 (**A**), RBD (**B**), and S2 (**C**) for 4 h (n = 4). Epithelial cells were also treated with the chemical macropinocytosis stimulator PMA (1 µM). (**D**) Cells were treated with EGF (0.5 nM, 4 h) ± EIPA (25 µM, 30 min pretreatment) in the presence of FITC-dextran (100 µg/mL) (n = 10). Internalization of FITC-dextran was quantified via FACS (ex. 493 nm, em. 518 nm). (**E**) Representative SEM images of epithelial cells treated with spike protein subunit S1, RBD, and S2 (1 and 10 µg/mL, 30 min) (n = 3). Data are presented as means ± SD. * *p* < 0.05; ** *p* < 0.005. P values were calculated using one way ANOVA with Tukey’s test for multiple comparisons. Scale bars are 10 µm.

SARS-CoV-2 is primarily transmitted via respiratory droplets and aerosols, which can be inhaled through the nose, leading to the infection of epithelial cells in the respiratory tract [5]. Within the gas exchange portion of the lungs, alveolar epithelial cells are the first cell type exposed to the virus and have been shown to be a major target in COVID-19 pneumonia [5]. Given this information, we investigated the ability of SARS-CoV-2 spike protein subunits to stimulate macropinocytosis in human alveolar epithelial cells. Incubation of cells with S1, RBD, or S2 did not stimulate internalization of FITC-dextran (0.001–10 µg/mL, 4 h) (Figure 3A–C). Similarly, chemical stimulation of macropinocytosis using PMA had no effect on dextran internalization by human alveolar epithelial cells (Figure 3A–C). Previous studies demonstrated macropinocytosis stimulation in alveolar epithelial cells in response to treatment with growth factors, including epidermal growth factor (EGF) [29]. To confirm that these cells are capable of macropinocytosis and exclude possible technical issues with the macropinocytosis assay, we next treated alveolar epithelial cells with EGF and quantified FITC-dextran internalization using flow cytometry. As shown in Figure 3D, EGF (0.5 nM, 4 h) stimulated FITC-dextran internalization. Preincubation of alveolar epithelial cells with EIPA inhibited EGF-stimulated FITC-dextran uptake, demonstrating that the uptake is mediated by macropinocytosis stimulation. Confirming our flow cytometry results, SEM imaging demonstrates that spike proteins do not stimulate membrane ruffle formation on the surface of human alveolar epithelial cells (Figure 3E).

### 3.4. Inhibition of Protein Kinase C, Phosphoinositide 3-Kinase and Nox2 Blocks SARS-CoV-2 Spike-Protein-Induced Macropinocytosis in Macrophages

**Figure 4:**
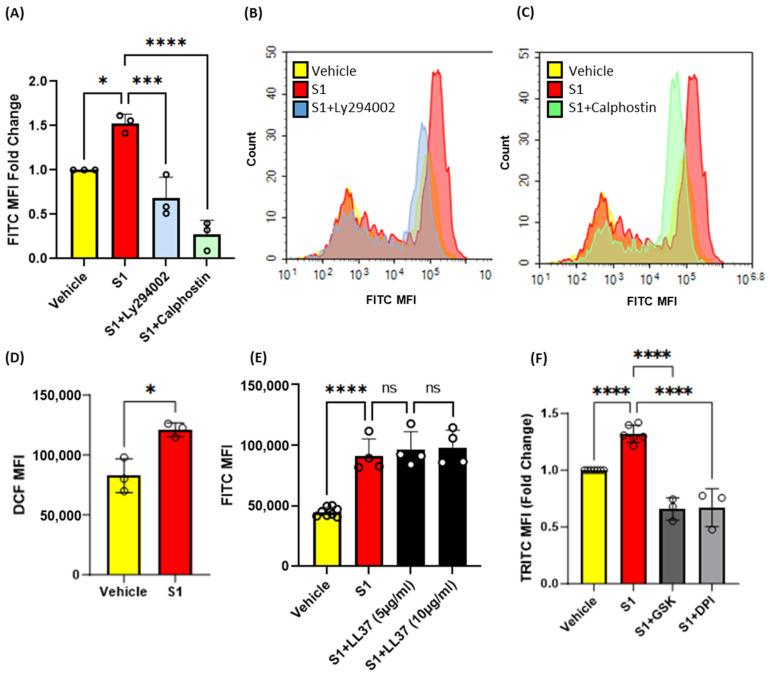
Pharmacological blockade of PKC, PI3K, and NOX2 inhibits SARS-CoV-2 spike protein-induced macropinocytosis in macrophages. (**A**) Murine bone marrow-derived macrophages were incubated with FITC-dextran (100 µg/mL) and treated with vehicle (PBS) or S1 (1 µg/mL, 4 h) ± LY394004 (10 µm, 30 min pretreatment) or Calphostin c (1 µm, 30 min pretreatment). Internalization of FITC-dextran was quantified via FACS (ex. 493 nm, em. 518 nm) (n = 3). (**B**,**C**) Representative histograms showing inhibitory effects of LY294004 (B) and Calphostin c (**C**). (**D**) Quantification of intracellular reactive oxygen species in cells treated with S1 (1 µg/mL, 1 h) using H_2_DCFDA (5 µM, 30 min) (n = 3). (**E**) Human primary PBMC-derived macrophages were pretreated with LL37 (5 µg/mL and 10 µg/mL, 30 min) and treated with S1 (1 µg/mL, 4 h). Internalization of FITC-dextran was quantified via FACS. (**F**) Cells were pretreated with GSK2795039 (20 µg/mL, 30 min), or DPI (5 µM, 30 min) and treated with S1 (1 µg/mL, 4 h) in the presence of TRITC-dextran (100 µg/mL). Internalization of TRITC-dextran was quantified via FACS (ex. 550 nm, em. 577 nm). Data are presented as means ± SD. ns = not significant. * *p* < 0.05; *** *p* < 0.001; **** *p* < 0.0001. P values were calculated using t-test or one-way ANOVA with Tukey’s test for multiple comparisons.

Previous studies have shown that protein kinase C (PKC) [20] and phosphoinositide 3-kinase (PI3K) [18] promote macropinocytosis in macrophages in response to growth factors and other stimulants. Next, macrophages were pretreated with vehicle, the PKC inhibitor calphostin c (1 µM, 30 min) or the PI3K inhibitor LY294002 (10 µM, 30 min), stimulated with S1 (1 µg/mL, 4 h), and the uptake of FITC-dextran (100 µg/mL, 4 h) was analyzed via flow cytometry. Our results demonstrated that inhibition of PKC and PI3K blocks SARS-CoV-2 S1-induced macropinocytosis (Figure 4A–C). Increased Nox2 activity has been linked to membrane ruffle formation and macropinocytic solute internalization via increased production of ROS [18]. PKC and PI3K serve as signal transduction mediators that promote Nox2 activation [18,20,30]. Nox2-derived ROS have been shown to stimulate macropinocytosis in response to the PKC activator PMA [18]. To further investigate the mechanism of spike-protein-induced macropinocytosis, we quantified S1-induced DCF fluorescence in macrophages using H_2_DCFDA, a cell permeant indicator for ROS. The cells were treated with S1 subunit (1 µg/mL, 1 h), incubated with H_2_DCFDA (5 µM, 30 min), and analyzed using flow cytometry. We observed significantly increased ROS production in response to S1 treatment (Figure 4D). To determine if Nox2 activity is required for macropinocytosis stimulation, murine bone marrow macrophages were pretreated with the flavoenzyme inhibitor DPI (5 µM, 30 min, non-specific Nox inhibitor) or selective Nox2 inhibitor GSK2795039 (20 µg/mL, 30 min) and stimulated with the S1 subunit in the presence of TRITC-dextran. Flow cytometry analysis showed that inhibition of Nox2 blocked macropinocytotic uptake of TRITC-dextran (Figure 4F) To determine if ACE2 binding is required for macropinocytosis stimulation of SARS-CoV-2 spike proteins, human PBMC-derived macrophages were pretreated with the peptidic ACE2 inhibitor LL37 (5 µg/mL and 10 µg/mL, 30 min) and stimulated with the S1 subunit (1 µg/mL, 4 h). As shown in Figure 4E, the pretreatment of human macrophages did not inhibit S1-induced stimulation of macropinocytosis. These results suggest that SARS-CoV-2 spike protein subunits stimulate macropinocytosis via PKC, PI3k, and Nox2 signaling. Further, our pharmacological study using LL37 suggests that the stimulation may be independent of ACE2.

### 3.5. SARS-CoV-2 Spike Proteins Stimulate Macrophage Macropinocytosis In Vivo

**Figure 5:**
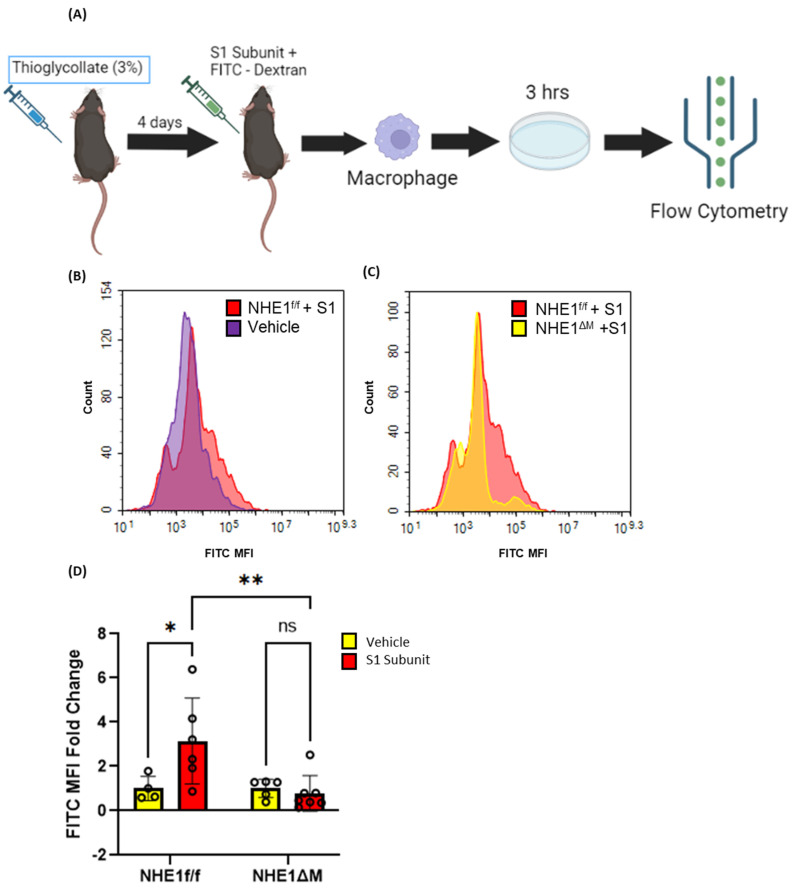
Recombinant SARS-CoV-2 spike protein stimulates macrophage macropinocytosis in vivo. (**A**) Schematic flow chart of experimental design. Representative FITC-dextran histograms indicating in vivo stimulation of macropinocytosis with S1 (**B**) and genetic inhibition of S1-induced macropinocytosis (**C**). (**D**) Bar graph indicates FITC-dextran MFI fold change in peritoneal macrophages of vehicle- or S1 (20 µg, 4 h)-treated NHE1^f/f^ and NHE1^ΔM^ mice (n = 4–7). Data are presented as means ± SD. ns = not significant. * *p* < 0.05; ** *p* < 0.005. P values were calculated using two-way ANOVA with Tukey’s test for multiple comparisons.

Next, we evaluated the ability of SARS-CoV-2 spike protein S1 subunit to stimulate macropinocytosis in peritoneal macrophages in vivo. Four days after thioglycolate (3%, 1 mL/30 g) injection, NHE1^ΔM^ mice and NHE1^f/f^ controls were treated with the spike protein S1 subunit (20 µg/30 g) and FITC-dextran (1 mg/30 g) via intraperitoneal (i.p.) administration (Figure 5A). Peritoneal macrophages were isolated after 4 h and processed for flow cytometry analysis. S1 protein stimulated FITC-dextran internalization in NHE1^f/f^ controls, but not in macropinocytosis-deficient NHE1^ΔM^ macrophages, in the peritoneal cavity in vivo (Figure 5B–D). These results demonstrate that SARS-CoV-2 S1 protein stimulates macropinocytosis in murine macrophages in vivo.

## 4. Discussion

Understanding the mechanism of SARS-CoV-2 entry into cells relevant to COVID-19 ARDS is important in developing therapies to combat the COVID-19 pandemic. While SARS-CoV-2 largely depends on angiotensin-converting enzyme 2 (ACE2) to enter cells, studies suggesting ACE2-independent mechanisms of entry have also been published [11,12]. Macropinocytosis is a receptor-independent and actin-dependent mode of endocytosis that mediates the internalization of extracellular fluid and pericellular solutes in various cell types [13]. Viruses including HIV-1 [15], Ebola virus [16], and herpes simplex virus 1 (HSV1) [17] utilize macropinocytosis to gain entry into cells. Within the coronavirus family, murine hepatitis virus (MHV) and SARS-CoV-1 have been shown to induce macropinocytosis but their entry into cells via this mechanism is limited [31,32].

The distinguishing feature of coronaviruses is the presence of spike proteins, which facilitates viral entry into cells via binding to specific host receptors. Coronavirus spike proteins consist of the S1 and S2 subunits. The binding of the virus to cells requires the S1 subunit, which contains a receptor-binding domain (RBD). Once bound, proteases cleave the spike protein subunits, which exposes and allows the S2 subunit to promote the fusion of the virus to cells [33]. Although largely similar, research has shown notable differences in the structure and functions of spike proteins within the coronavirus family that may explain different infection rates. For example, the receptor-binding domain of murine hepatitis virus (MHV) lies on the N-terminal of the S1 subunit and binds to the protein receptor CEACAM1a [34]. In SARS-CoV and SARS-CoV-2, this domain lies on the C-terminal and allows the virus to bind to ACE2 [35]. The presence of the RBD on the C-terminal also allows the spike proteins to change conformations, promoting dissociation between the two subunits. Furthermore, SARS-CoV-2 spike proteins are unique because they contain an additional cleavage site, which has been shown to enhance infection [36]. The specific mechanisms explaining why MHV or SARS-CoV-1 entry into cells via macropinocytosis is limited are currently unknown, but the above-described structural and functional differences in spike proteins may explain differences in infection rate and host cell entry.

To our knowledge, no previous studies have investigated the ability of SARS-CoV-2 spike proteins to stimulate macropinocytosis in cell types directly relevant to SARS-CoV-2-associated lung infection and pneumonia. Interestingly, a recent study showed that SARS-CoV-2 promotes macropinocytosis in kidney and colon epithelial cells [12]. We initially tested our hypothesis on alveolar epithelial cells because this is the first cell type exposed to SARS-CoV-2 and these cells have been shown to be a major target in COVID-19 pneumonia [37]. In epithelial cells, SARS-CoV-2 has been shown to enter cells primarily via spike protein-ACE2 interaction. The binding of the viral spike protein to the host ACE2 receptor promotes fusion between the virus and the host cell and ultimately leads to the internal transfer of viral RNA. Within the cell, ribosomes translate the viral RNA into polyproteins. These proteins assist in the generation of new virions, which are assembled in the ER and Golgi and released out of the cell [38]. Infected epithelial cells have been shown to release proinflammatory cytokines, which contribute to alveolar damage and the development of ARDS [39].

The effect of SARS-CoV-2 on macrophages is less clear. Infected macrophages have been found in the lungs, spleen, lymph nodes, and the cardiovascular system in deceased patients with COVID-19 [40,41,42]. Single cell RNA-seq and ex vivo studies with lung explants have shown that within SARS-CoV-2-infected lungs, both ACE2-positive and ACE2-negative macrophages are present, but the expression of ACE2 is significantly lower than in alveolar epithelial cells [43]. Nevertheless, many macrophages have shown to be positive for SARS-CoV-2 RNA or protein in vivo [44,45], suggesting an ACE2-independent mechanism of viral entry into macrophages. Recent studies have shown that SARS-CoV-2 can infect host cells via clathrin-mediated endocytosis [46], caveolae-dependent endocytosis [47], phagocytosis [48], and macropinocytosis [12] to initiate an inflammatory cascade. The cell-specific mechanisms of infection and their consequences are still unknown and are a future area of research in the laboratory.

Lung macrophages are considered early immune defenders against respiratory pathogens. Their defensive activities include antigen recognition and presentation, promoting inflammation, activating adaptive immune cells, and contributing to tissue repair [49]. Studies have shown that while SARS-CoV-2 infection in macrophages does not lead to viral replication, it does impair the defensive ability of macrophages and may dysregulate their functions, leading to cytokine storm and tissue damage [43]. Interestingly, studies have shown that lung tissue resident alveolar macrophages are depleted in COVID-19 and replaced by monocyte-derived macrophages in a phenomenon known as “macrophage disappearance reaction” [50]. These monocyte-derived macrophages are guided to the lungs by pro-inflammatory cytokines in a defense response due to the change in phenotype and impairment of function of resident alveolar macrophages [50].

Our FITC-dextran flow cytometry results demonstrate that neither SARS-CoV-2 spike protein subunit S1 nor S2 induces macropinocytosis in alveolar epithelial cells, but they do so in both murine and human macrophages. To confirm that the increase in internalized dextran was a result of macropinocytosis and not due to changes in pH, similar experiments were performed in the presence of TRITC-dextran, a pH-insensitive fluorescent probe. It is important to note that purchased recombinant S1, RBD, and S2 regions of the spike protein were made in E. coli, which contain lipopolysaccharides (LPS) on the cell surface. Studies have shown that LPS is a known inducer of macropinocytosis and membrane ruffling [51,52,53]. Therefore, polymyxin b, an inhibitor of LPS, was used to confirm that macropinocytosis is due to recombinant spike proteins and not a consequence of LPS contamination.

Since the RBD of S1 binds to human but not to mouse ACE2, the stimulatory effects in both mouse and human macrophages indicate that this process may occur independently from ACE2. Consistent with these results, the antimicrobial peptide LL37 that inhibits SARS-CoV-2 binding to human ACE2 did not block spike-protein-induced macropinocytosis in primary human macrophages [54]. A limitation of this study is that we did not use primary human alveolar macrophages, which would have been more relevant to COVID-19 pneumonia. Additionally, we did not investigate the ability of live SARS-CoV-2 to stimulate macropinocytosis in macrophages and its subsequent internalization.

The pharmacological blockade of NHE1 is currently regarded as the gold-standard approach to inhibit macropinocytosis in vitro and in vivo [27,28]. The inhibition of NHE1 blocks macropinocytosis by lowering the submembranous pH, thereby suppressing the activity of the small GTPase RAC1 and inhibiting actin remodeling required for the development of membrane protrusions and macropinosome formation. Our laboratory has recently demonstrated that selective deletion of the *Nhe1* gene in myeloid cells inhibits macrophage macropinocytosis [28]. It is important to note that this genetic model may have macropinocytosis-independent effects [55]. In the current study, we used both pharmacological (EIPA) and genetic (NHE1^ΔM^) approaches to investigate the ability of spike proteins to stimulate macropinocytosis in vitro and in vivo. As previously mentioned, ethyl-isopropyl amiloride (EIPA) is a specific and potent inhibitor of the sodium hydrogen exchanger and is currently considered the gold-standard inhibitor of macropinocytosis [27,28]. High-resolution scanning electron microscopy (SEM) is a commonly used imaging technique to visualize and quantify macropinocytosis-associated membrane ruffling in vitro [14]. Our SEM experiments demonstrate emerging membrane projections, fully formed dorsal ruffles and circularized membrane protrusions forming cups on the surface of human macrophages treated with SARS-CoV-2 spike protein subunits, thus confirming macropinocytosis stimulation.

Alveolar epithelial cells are the first point-of-contact for SARS-CoV-2 in the gas exchange compartment of the lungs and represent a major target for virus entry. Next, we tested whether the spike protein subunits stimulate macropinocytosis in human alveolar epithelial cells. Interestingly, SARS-CoV-2 spike proteins did not stimulate membrane ruffling or macropinocytic solute internalization in alveolar epithelial cells. Although we and other researchers have shown that epithelial cells are capable of macropinocytosis in response to epithelial growth factor (EGF) treatment [29], spike protein subunits failed to stimulate uptake of FITC-dextran and produced no membrane ruffles, suggesting that the ability of SARS-CoV-2 spike protein to stimulate macropinocytosis is cell type specific. The expression of EGF and its receptor is elevated in the bronchial epithelium of patients with chronic obstructive pulmonary disease (COPD) [56]. These results suggest that SARS-CoV-2 macropinocytosis may occur in alveolar epithelial cells in response to high EGF levels observed in COPD patients.

To investigate the mechanism of SARS-CoV-2 spike protein-induced macropinocytosis in macrophages, we conducted flow cytometry experiments using PKC, PI3K, and Nox2 inhibitors. Calphostin C [57] and Ly294002 [58] are both potent and selective inhibitors of PKC and PI3K, respectively. Diphenyleneiodoium chloride (DPI), a potent pan-inhibitor of NADPH oxidases [59,60], and GSK2795039, a novel and specific small molecule NOX2 inhibitor [61,62], were used to confirm the role of NOX2 in SARS-CoV-2 spike protein-induced macropinocytosis. PKC and PI3K have been shown to play important roles in macropinocytosis stimulation in response to treatment with growth factors and cytokines [18,20]. In addition, a recent study has shown that PI3K blockade inhibits SARS-CoV2-induced macropinocytosis in kidney epithelial cells [12]. Previous studies demonstrated that PKC and PI3K play important signal-transducing roles in Nox2 activation in macrophages [18,20]. The resulting production of ROS has been shown to promote membrane ruffling and macropinocytosis. Our results demonstrated that the blockade of PKC, PI3K, and Nox2 inhibits spike protein-induced macropinocytosis.

In conclusion, our results identify SARS-CoV-2 spike protein as a major stimulator of macropinocytosis in both murine and human macrophages, but not in alveolar epithelial cells. Although the development of COVID-19 vaccines has largely mitigated the disease mortality rate, long-term negative health outcomes remain a major issue, especially in high-risk individuals. Studies have shown that in some individuals with COVID-19, neurological, respiratory, gastrointestinal and cardiovascular symptoms can persist months or years after initial infection [63]. Importantly, cleaved SARS-CoV-2 spike proteins may cause cardiomyocyte inflammation and myocarditis [64] and induce endothelial cell dysfunction in the blood–brain barrier [65]. Patients with myocarditis have elevated levels of circulating full-length spike protein several weeks after vaccination. In addition, SARS-CoV-2 infects cells that lack ACE2, indicating that some human cells possess ACE2-independent entry mechanisms. Our study suggests that macropinocytosis stimulation may mediate alternative, ACE2-independent mechanisms of SARS-CoV-2 entry and infection [66]. Taken together, these results may contribute to a better understanding of SARS-CoV-2 infection and COVID-19 pathogenesis.

## Data Availability

The datasets generated for this study will be made available to interested researchers upon request.

## Supplementary Materials

The following supporting information can be downloaded at: https://www.mdpi.com/article/10.3390/antiox13020175/s1, Figure S1: Quantification of macrophage FITC-dextran uptake in response to recombinant SARS-CoV-2 spike protein treatment.

## References

[1] Collaborators C.-E.M. (2022). Estimating excess mortality due to the COVID-19 pandemic: A systematic analysis of COVID-19-related mortality, 2020–2021. Lancet.

[2] Hulswit R.J., de Haan C.A., Bosch B.J. (2016). Coronavirus Spike Protein and Tropism Changes. Adv. Virus Res..

[3] Zhou P., Yang X.L., Wang X.G., Hu B., Zhang L., Zhang W., Si H.R., Zhu Y., Li B., Huang C.L. (2020). A pneumonia outbreak associated with a new coronavirus of probable bat origin. Nature.

[4] Hoffmann M., Kleine-Weber H., Schroeder S., Kruger N., Herrler T., Erichsen S., Schiergens T.S., Herrler G., Wu N.H., Nitsche A. (2020). SARS-CoV-2 Cell Entry Depends on ACE2 and TMPRSS2 and Is Blocked by a Clinically Proven Protease Inhibitor. Cell.

[5] Bridges J.P., Vladar E.K., Huang H., Mason R.J. (2022). Respiratory epithelial cell responses to SARS-CoV-2 in COVID-19. Thorax.

[6] Lamers M.M., Haagmans B.L. (2022). SARS-CoV-2 pathogenesis. Nat. Rev. Microbiol..

[7] Konopka K.E., Nguyen T., Jentzen J.M., Rayes O., Schmidt C.J., Wilson A.M., Farver C.F., Myers J.L. (2020). Diffuse alveolar damage (DAD) resulting from coronavirus disease 2019 Infection is Morphologically Indistinguishable from Other Causes of DAD. Histopathology.

[8] Knoll R., Schultze J.L., Schulte-Schrepping J. (2021). Monocytes and Macrophages in COVID-19. Front. Immunol..

[9] Merad M., Martin J.C. (2020). Pathological inflammation in patients with COVID-19: A key role for monocytes and macrophages. Nat. Rev. Immunol..

[10] Grant R.A., Morales-Nebreda L., Markov N.S., Swaminathan S., Querrey M., Guzman E.R., Abbott D.A., Donnelly H.K., Donayre A., Goldberg I.A. (2021). Circuits between infected macrophages and T cells in SARS-CoV-2 pneumonia. Nature.

[11] Glebov O.O. (2020). Understanding SARS-CoV-2 endocytosis for COVID-19 drug repurposing. FEBS J..

[12] Zhang Y.Y., Liang R., Wang S.J., Ye Z.W., Wang T.Y., Chen M., Liu J., Na L., Yang Y.L., Yang Y.B. (2022). SARS-CoV-2 hijacks macropinocytosis to facilitate its entry and promote viral spike-mediated cell-to-cell fusion. J. Biol. Chem..

[13] Lin X.P., Mintern J.D., Gleeson P.A. (2020). Macropinocytosis in Different Cell Types: Similarities and Differences. Membranes.

[14] Ahn W., Singla B., Marshall B., Csanyi G. (2021). Visualizing Membrane Ruffle Formation using Scanning Electron Microscopy. J. Vis. Exp..

[15] Marechal V., Prevost M.C., Petit C., Perret E., Heard J.M., Schwartz O. (2001). Human immunodeficiency virus type 1 entry into macrophages mediated by macropinocytosis. J. Virol..

[16] Aleksandrowicz P., Marzi A., Biedenkopf N., Beimforde N., Becker S., Hoenen T., Feldmann H., Schnittler H.J. (2011). Ebola virus enters host cells by macropinocytosis and clathrin-mediated endocytosis. J. Infect. Dis..

[17] Nicola A.V., Hou J., Major E.O., Straus S.E. (2005). Herpes simplex virus type 1 enters human epidermal keratinocytes, but not neurons, via a pH-dependent endocytic pathway. J. Virol..

[18] Ghoshal P., Singla B., Lin H., Feck D.M., Cantu-Medellin N., Kelley E.E., Haigh S., Fulton D., Csanyi G. (2017). Nox2-Mediated PI3K and Cofilin Activation Confers Alternate Redox Control of Macrophage Pinocytosis. Antioxid. Redox Signal.

[19] Bedard K., Krause K.H. (2007). The NOX family of ROS-generating NADPH oxidases: Physiology and pathophysiology. Physiol. Rev..

[20] Singla B., Lin H.P., Ghoshal P., Cherian-Shaw M., Csanyi G. (2019). PKCdelta stimulates macropinocytosis via activation of SSH1-cofilin pathway. Cell Signal.

[21] Yoshida S., Gaeta I., Pacitto R., Krienke L., Alge O., Gregorka B., Swanson J.A. (2015). Differential signaling during macropinocytosis in response to M-CSF and PMA in macrophages. Front. Physiol..

[22] Bryant D.M., Kerr M.C., Hammond L.A., Joseph S.R., Mostov K.E., Teasdale R.D., Stow J.L. (2007). EGF induces macropinocytosis and SNX1-modulated recycling of E-cadherin. J. Cell Sci..

[23] Yoshida S., Pacitto R., Yao Y., Inoki K., Swanson J.A. (2015). Growth factor signaling to mTORC1 by amino acid-laden macropinosomes. J. Cell Biol..

[24] Zhang X., Goncalves R., Mosser D.M. (2008). The isolation and characterization of murine macrophages. Curr. Protoc. Immunol..

[25] Tardelli M., Zeyda K., Moreno-Viedma V., Wanko B., Grun N.G., Staffler G., Zeyda M., Stulnig T.M. (2016). Osteopontin is a key player for local adipose tissue macrophage proliferation in obesity. Mol. Metab..

[26] Kruth H.S., Jones N.L., Huang W., Zhao B., Ishii I., Chang J., Combs C.A., Malide D., Zhang W.Y. (2005). Macropinocytosis is the endocytic pathway that mediates macrophage foam cell formation with native low density lipoprotein. J. Biol. Chem..

[27] Jayashankar V., Edinger A.L. (2020). Macropinocytosis confers resistance to therapies targeting cancer anabolism. Nat. Commun..

[28] Lin H.P., Singla B., Ahn W., Ghoshal P., Blahove M., Cherian-Shaw M., Chen A., Haller A., Hui D.Y., Dong K. (2022). Receptor-independent fluid-phase macropinocytosis promotes arterial foam cell formation and atherosclerosis. Sci. Transl. Med..

[29] West M.A., Bretscher M.S., Watts C. (1989). Distinct endocytotic pathways in epidermal growth factor-stimulated human carcinoma A431 cells. J. Cell Biol..

[30] Singla B., Ghoshal P., Lin H., Wei Q., Dong Z., Csanyi G. (2018). PKCdelta-Mediated Nox2 Activation Promotes Fluid-Phase Pinocytosis of Antigens by Immature Dendritic Cells. Front. Immunol..

[31] Wang H., Yang P., Liu K., Guo F., Zhang Y., Zhang G., Jiang C. (2008). SARS coronavirus entry into host cells through a novel clathrin- and caveolae-independent endocytic pathway. Cell Res..

[32] Freeman M.C., Peek C.T., Becker M.M., Smith E.C., Denison M.R. (2014). Coronaviruses induce entry-independent, continuous macropinocytosis. mBio.

[33] Huang Y., Yang C., Xu X.F., Xu W., Liu S.W. (2020). Structural and functional properties of SARS-CoV-2 spike protein: Potential antivirus drug development for COVID-19. Acta Pharmacol. Sin..

[34] Miura H.S., Nakagaki K., Taguchi F. (2004). N-terminal domain of the murine coronavirus receptor CEACAM1 is responsible for fusogenic activation and conformational changes of the spike protein. J. Virol..

[35] Zhou R., Zeng R., von Brunn A., Lei J. (2020). Structural characterization of the C-terminal domain of SARS-CoV-2 nucleocapsid protein. Mol. Biomed..

[36] Wang Z., Zhong K., Wang G., Lu Q., Li H., Wu Z., Zhang Z., Yang N., Zheng M., Wang Y. (2023). Loss of furin site enhances SARS-CoV-2 spike protein pseudovirus infection. Gene.

[37] Mulay A., Konda B., Garcia G., Yao C., Beil S., Villalba J.M., Koziol C., Sen C., Purkayastha A., Kolls J.K. (2021). SARS-CoV-2 infection of primary human lung epithelium for COVID-19 modeling and drug discovery. Cell Rep..

[38] V’Kovski P., Kratzel A., Steiner S., Stalder H., Thiel V. (2021). Coronavirus biology and replication: Implications for SARS-CoV-2. Nat. Rev. Microbiol..

[39] Zheng J., Miao J., Guo R., Guo J., Fan Z., Kong X., Gao R., Yang L. (2022). Mechanism of COVID-19 Causing ARDS: Exploring the Possibility of Preventing and Treating SARS-CoV-2. Front. Cell Infect. Microbiol..

[40] Abdullaev A., Odilov A., Ershler M., Volkov A., Lipina T., Gasanova T., Lebedin Y., Babichenko I., Sudarikov A. (2021). Viral Load and Patterns of SARS-CoV-2 Dissemination to the Lungs, Mediastinal Lymph Nodes, and Spleen of Patients with COVID-19 Associated Lymphopenia. Viruses.

[41] Matveeva O., Nechipurenko Y., Lagutkin D., Yegorov Y.E., Kzhyshkowska J. (2022). SARS-CoV-2 infection of phagocytic immune cells and COVID-19 pathology: Antibody-dependent as well as independent cell entry. Front. Immunol..

[42] Jum’ah H., Kundrapu S., Jabri A., Kondapaneni M., Tomashefski J.F., Loeffler A.G. (2022). Cardiac macrophage density in Covid-19 infection: Relationship to myocyte necrosis and acute lung injury. Cardiovasc. Pathol..

[43] Labzin L.I., Chew K.Y., Eschke K., Wang X., Esposito T., Stocks C.J., Rae J., Patrick R., Mostafavi H., Hill B. (2023). Macrophage ACE2 is necessary for SARS-CoV-2 replication and subsequent cytokine responses that restrict continued virion release. Sci. Signal.

[44] Bost P., Giladi A., Liu Y., Bendjelal Y., Xu G., David E., Blecher-Gonen R., Cohen M., Medaglia C., Li H. (2020). Host-Viral Infection Maps Reveal Signatures of Severe COVID-19 Patients. Cell.

[45] Delorey T.M., Ziegler C.G.K., Heimberg G., Normand R., Yang Y., Segerstolpe A., Abbondanza D., Fleming S.J., Subramanian A., Montoro D.T. (2021). COVID-19 tissue atlases reveal SARS-CoV-2 pathology and cellular targets. Nature.

[46] Bayati A., Kumar R., Francis V., McPherson P.S. (2021). SARS-CoV-2 infects cells after viral entry via clathrin-mediated endocytosis. J. Biol. Chem..

[47] Knyazev E., Nersisyan S., Tonevitsky A. (2021). Endocytosis and Transcytosis of SARS-CoV-2 Across the Intestinal Epithelium and Other Tissue Barriers. Front. Immunol..

[48] Garcia-Nicolas O., Godel A., Zimmer G., Summerfield A. (2023). Macrophage phagocytosis of SARS-CoV-2-infected cells mediates potent plasmacytoid dendritic cell activation. Cell Mol. Immunol..

[49] Hirayama D., Iida T., Nakase H. (2017). The Phagocytic Function of Macrophage-Enforcing Innate Immunity and Tissue Homeostasis. Int. J. Mol. Sci..

[50] Bain C.C., Lucas C.D., Rossi A.G. (2022). Pulmonary macrophages and SARS-Cov2 infection. Int. Rev. Cell Mol. Biol..

[51] Quinn S.E., Huang L., Kerkvliet J.G., Swanson J.A., Smith S., Hoppe A.D., Anderson R.B., Thiex N.W., Scott B.L. (2021). The structural dynamics of macropinosome formation and PI3-kinase-mediated sealing revealed by lattice light sheet microscopy. Nat. Commun..

[52] Redka D.S., Gutschow M., Grinstein S., Canton J. (2018). Differential ability of proinflammatory and anti-inflammatory macrophages to perform macropinocytosis. Mol. Biol. Cell.

[53] Condon N.D., Heddleston J.M., Chew T.L., Luo L., McPherson P.S., Ioannou M.S., Hodgson L., Stow J.L., Wall A.A. (2018). Macropinosome formation by tent pole ruffling in macrophages. J. Cell Biol..

[54] Li D., Chen P., Shi T., Mehmood A., Qiu J. (2021). HD5 and LL-37 Inhibit SARS-CoV and SARS-CoV-2 Binding to Human ACE2 by Molecular Simulation. Interdiscip. Sci..

[55] Liu C.L., Zhang X., Liu J., Wang Y., Sukhova G.K., Wojtkiewicz G.R., Liu T., Tang R., Achilefu S., Nahrendorf M. (2019). Na(+)-H(+) exchanger 1 determines atherosclerotic lesion acidification and promotes atherogenesis. Nat. Commun..

[56] Saied E.M., Bediwy A.S. (2011). Expression of epidermal growth factor receptor (EGFR) in the bronchial epithelium of patients with chronic obstructive pulmonary disease (COPD). Eur. Respir. J..

[57] Kobayashi E., Nakano H., Morimoto M., Tamaoki T. (1989). Calphostin C (UCN-1028C), a novel microbial compound, is a highly potent and specific inhibitor of protein kinase C. Biochem. Biophys. Res. Commun..

[58] Gharbi S.I., Zvelebil M.J., Shuttleworth S.J., Hancox T., Saghir N., Timms J.F., Waterfield M.D. (2007). Exploring the specificity of the PI3K family inhibitor LY294002. Biochem. J..

[59] Piszczatowska K., Przybylska D., Sikora E., Mosieniak G. (2020). Inhibition of NADPH Oxidases Activity by Diphenyleneiodonium Chloride as a Mechanism of Senescence Induction in Human Cancer Cells. Antioxidants.

[60] Altenhofer S., Radermacher K.A., Kleikers P.W., Wingler K., Schmidt H.H. (2015). Evolution of NADPH Oxidase Inhibitors: Selectivity and Mechanisms for Target Engagement. Antioxid. Redox Signal.

[61] Hirano K., Chen W.S., Chueng A.L., Dunne A.A., Seredenina T., Filippova A., Ramachandran S., Bridges A., Chaudry L., Pettman G. (2015). Discovery of GSK2795039, a Novel Small Molecule NADPH Oxidase 2 Inhibitor. Antioxid. Redox Signal.

[62] de Oliveira M.G., Monica F.Z., Passos G.R., Victorio J.A., Davel A.P., Oliveira A.L.L., Parada C.A., D’Ancona C.A.L., Hill W.G., Antunes E. (2022). Selective Pharmacological Inhibition of NOX2 by GSK2795039 Improves Bladder Dysfunction in Cyclophosphamide-Induced Cystitis in Mice. Antioxidants.

[63] Davis H.E., McCorkell L., Vogel J.M., Topol E.J. (2023). Long COVID: Major findings, mechanisms and recommendations. Nat. Rev. Microbiol..

[64] Bozkurt B. (2023). Shedding Light on Mechanisms of Myocarditis With COVID-19 mRNA Vaccines. Circulation.

[65] Petrovszki D., Walter F.R., Vigh J.P., Kocsis A., Valkai S., Deli M.A., Der A. (2022). Penetration of the SARS-CoV-2 Spike Protein across the Blood-Brain Barrier, as Revealed by a Combination of a Human Cell Culture Model System and Optical Biosensing. Biomedicines.

[66] Lim S., Zhang M., Chang T.L. (2022). ACE2-Independent Alternative Receptors for SARS-CoV-2. Viruses.

