# Supplementary material for: SARS-CoV-2 Spike Protein Stimulates Macropinocytosis in Murine and Human Macrophages via PKC-NADPH Oxidase Signaling
Source: Antioxidants (Basel). 2024 Jan 30;13(2):175. doi: 10.3390/antiox13020175 (PMC10885885; doi:10.3390/antiox13020175)
Supplement: Supplementary file 1 [file antioxidants-13-00175-s001.zip › antioxidants-2798353-supplementary.pdf]

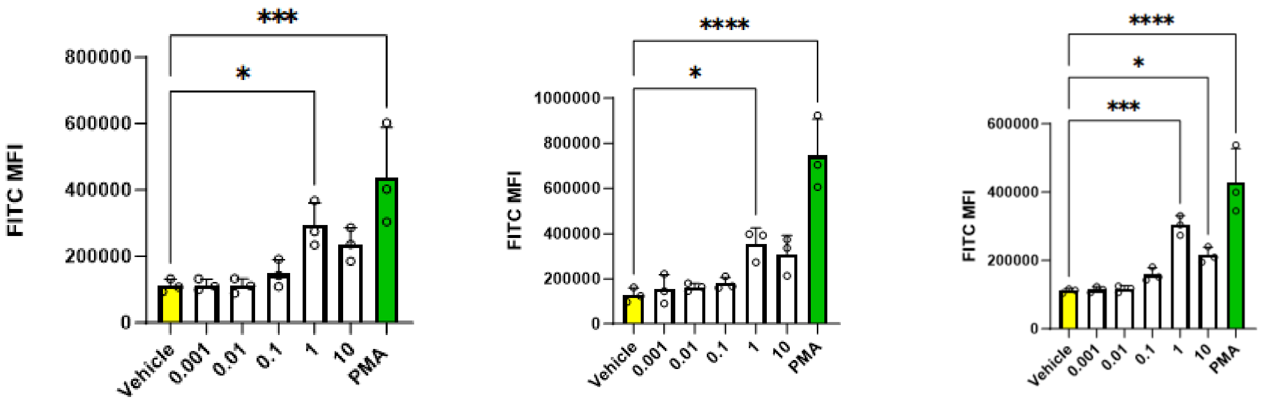

Supplementary Figure S1. Quantification of macrophage FITC-dextran uptake in response to Recombinant SARS-CoV-2 spike protein treatment. Murine bone marrow-derived macrophages were incubated with FITC-dextran (100 µg/ml) and treated with vehicle (PBS) or with different concentrations (0.001 µg/ml – 10 µg/ml) of the spike protein subunits S1 **(A)**, RBD **(B)**, and S2 **(C)** for 4 hours. Data are presented as means ± SD. *P<0.05; ***P<0.001; ****P<0.0001. P values were calculated using one-way ANOVA with Tukey's test for multiple comparisons.
